# Therapeutic Implementation of Oncolytic Viruses for Cancer Immunotherapy: Review of Challenges and Current Clinical Trials

**DOI:** 10.36266/JBSR/164

**Published:** 2022-10-20

**Authors:** X Wang, HM Maeng, J Lee, C Xie

**Affiliations:** a Thoracic and GI Malignancies Branch, Center for Cancer Research, National Cancer Institute, National Institutes of Health, Bethesda, MD 20814, USA; b Vaccine Branch, Center for Cancer Research, National Cancer Institute, National Institutes of Health, Bethesda, MD 20814, USA

**Keywords:** Oncolytic virus, Cancer, vaccinia virus, Adenovirus, Herpes simplex virus, Vaccinia virus, Newcastle disease virus, Poliovirus, Tumor stroma

## Abstract

The development of cancer therapeutics has evolved from general targets with radiation and chemotherapy and shifted toward treatments with a more specific mechanism of action such as small molecule kinase inhibitors, monoclonal antibodies against tumor antigens, or checkpoint inhibitors. Recently, oncolytic viruses (OVs) have come to the forefront as a viable option for cancer immunotherapy, especially for “cold” tumors, which are known to inhabit an immunologically suppressive tumor microenvironment. Desired characteristics of viruses are selected through genetic attenuation of uncontrolled virulence, and some genes are replaced with ones that enhance conditional viral replication within tumor cells. Treatment with OVs must overcome various hurdles such as premature viral suppression by the host’s immune system and the dense stromal barrier. Currently, clinical studies investigate the efficacy of OVs in conjunction with various anti-cancer therapeutics, including radiotherapy, chemotherapy, immune checkpoint inhibitors, and monoclonal antibodies. Thus, future research should explore how cancer therapeutics work synergistically with certain OVs in order to create more effective combination therapies and improve patient outcomes.

## Introduction

Cancer continues to be one of leading causes of death and remains a major threat to human health. The World Health Organization predicts the rate of cancer incidence and mortality will continue to rise in the next 20 years [1]. The effects of traditional therapeutic modalities such as surgical resection, chemotherapy, radiation therapy, and recently developed immunotherapy are not optimal despite recent improvements. Thus, there is a critical need for novel anti-tumoral strategies.

Vaccines have become an important milestone in the development of the field of immunology and success in healthcare. The basic premise lies with inoculation of an attenuated form or noninfectious portion of the infectious organism into the body to elicit an immune response. Vaccines grant protective immunity to the body by program the immune system to recognize and target these foreign invaders. When exposed to the nonimpaired version of a virus, the immune system is then able to respond quickly and efficiently to subdue the virus, thus preventing major infection [2]. As a result, vaccine development has been critical in drastically reducing the number of deaths due to smallpox, yellow fever [3], measles, mumps, rubella, and varicella [4]. Certain viruses have been recognized for the ability to target tumor cells. These oncolytic viruses (OVs) tend to use live and infectious viruses and have become a topic of interest in the arena of cancer therapeutics because of their ability to induce selective cell death and specific anti-tumor immunity. In this review, we summarize and integrate what has been published in the literature in terms of the wide diversity of OVs, discuss the challenges in oncolytic viral therapy, and suggest how modification and implementation of OVs in conjunction with traditional cancer therapies may enhance the overall success of adjuvant treatments.

## Characteristic of Oncolytic Virus

OVs are a type of cancer therapy where viruses are selected for their oncolytic capacity. Often, these viruses are attenuated through alterations in the viral genome that allow for reduced cytotoxicity toward non-cancerous cells and conditional replication in cancer cells. Alternatively, OVs may also be selected for through mutliple passages in tumor tissues. Key viral genes needed for virulence are substituted with genes that encode proteins to specifically target tumor cells. Thus, this strategy prevents viral targeting of nonmalignant tissues and restricts viral replication to only within tumor cells [5,6]. Moreover, use of engineered viruses in virotherapy often comes with a question of potential insertional mutagenesis where the viral genome integrates itself into the host’s genome [7]. However, OVs undergo multiple preclinical studies which assess for efficacy and safety before application in humans as demonstrated by Duerner et al.’s (2008) study of conditionally replication-competent murine leukaemia virus [8]. Despite the low possibility of genomic integration and detrimental impact in the clinical outcome, concrete long term safety data regarding administration of these engineered viruses in humans is needed as more therapeutics are implementing these viruses in both oncology and non-oncology clinics [7].

This selective elimination of cancer cells often depends on the viral strain, cancer type, tumor microenvironment (TME), and host immune system. OVs are intended to preferentially target cancer cells by exploiting unique extracellular surface markers on cancer cell, thus gaining entry into the cell. Commonly overexpressed surface markers in tumor cells are CD46, CD155, and integrin α2β1, which serve as receptors for measles virus, poliovirus, and echovirus respectively [9,10]. Cancer cells often have specific mutations (i.e., aberrations with in BCL-2, EGFR, PTEN, RAS, RB1, TP53, and WNT) that allow for unregulated cell proliferation. However, these mutations may also predispose the tumor cells to viral infection and subsequent cytotoxic elimination [11–13]. Nevertheless, normal cells often induce interferon (IFN) expression in response to viral infection. However, due to the inability of cancer cells to to induce type 1 IFN signaling [11], OVs are able to freely replicate within cancer cells, subsequently inducing oncolysis and release of viral progeny to continue the infection cycle. In addition, OVs may be armed to express immunostimulatory cytokines/chemokines (e.g., tumor necrotic factor (TNF), Interferon (IFN) α, and granulocyte-macrophage colony-stimulating factor (GM-CSF)), which allow the viruses to elicit a strong host immune response [3,4].

OV treatment begins with inoculation of the virus followed by viral replication which generates excessive virus-induced damage, compromising the integrity of cancer cells and results in oncolysis [11]. OV replication has also been found to promote strong anti-tumor immunity through the induction of immunogenic cell death (ICD), which releases tumor antigens (TA), damage-associated molecular patterns (DAMPs), OV-derived pathogen-associated molecular patterns (PAMPs), and inflammatory cytokines to activate and recruit both innate and adaptive immune cells [14,15]. PAMPs function to alert the immune system of the presence of pathogens [16], while DAMPs function to bring awareness to tissue trauma by binding to corresponding receptors on dendritic cells to induce T-cell activation and strongly influences the immune balance in the TME [17,18].

**Figure 1.**
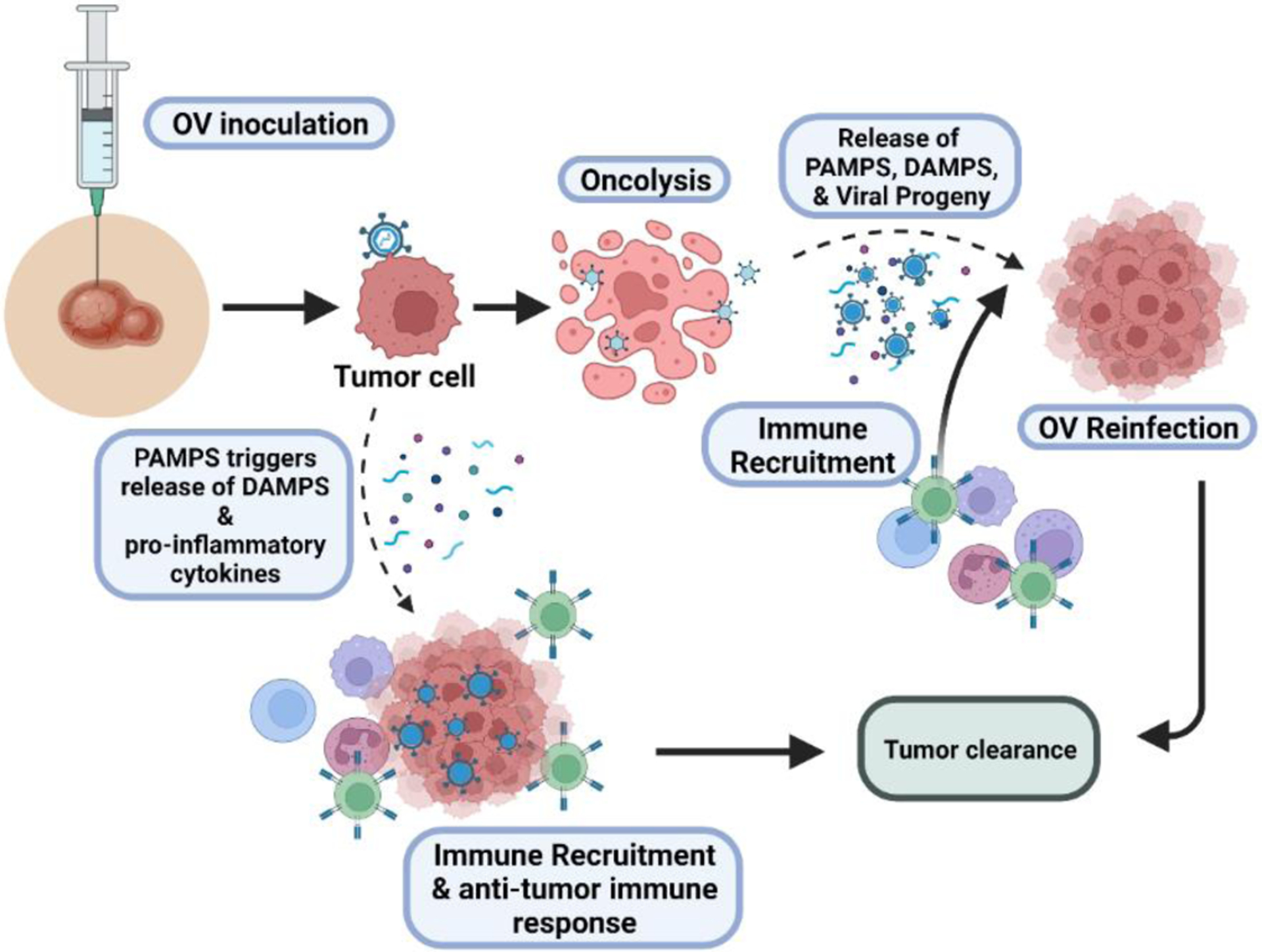
Overview of the pathway in oncolytic virotherapy. Inoculation introduces the oncolytic virus (OV) to the tumor. OVs bind to specific extracellular surface markers that are solely expressed on tumor cells, gaining entry into the cell. Hijacking the host machinery, the OVs rapidly replicate and induce oncolysis, releasing viral progeny, pathogen-associated molecular patterns (PAMPs), damage-associated molecular patterns (DAMPs), chemokines, and cytokines. Released viral progeny continue the oncolytic cycle by binding to neighboring tumor cells, while the other factors work to recruit various types of immune cells (e.g., CD4+ T cells, CD8+ T cells, and NK cells) to the tumor, allowing for tumor infiltration and enhanced eradication of malignant cells. Alternatively, some oncolytic viruses do not induce oncolysis, rather these viruses induce secretion of DAMPS and pro-inflammatory cytokines to recruit immune cells to target the tumor cells. Created with BioRender.com.

Furthermore, some OVs trigger the anti-tumor response without viral replication-mediated oncolysis. Binding of OVs to the tumor cell triggers activation of an antiviral immune response, where PAMPS trigger secretion of cytokine, and DAMPS to recruit immune cells to the area. Thus, this alternative pathway also promotes an anti-tumor immune response (Figure 1) [19,20]. Therefore, OVs are a feasible option to target notoriously non-immunogenic “cold” tumors, which are known to inhabit a TME that suppresses immune responses and T cell invasion by effectively stimulating both the innate and adaptive immune system. Many of these cold tumors are also non-reponsive to current available immune checkpoint inhibitors and demonstrates in crutial need improvement in the efficacy of cancer immunotherapy [21,22].

### Oncolytic Virus Strains

Various OVs with anti-tumor properties have been explored, including both DNA and RNA viruses (Table 1). It is important to note that not all DNA/RNA viruses are oncolytic viruses. The important factor that separates oncolytic viruses from other genetically altered viruses for treatment purposes is that OVs are able to replicate and induce cell lysis, hence their name. Genetically altered viruses such as certain adenovirus agents serve as viral vectors that simply deliver the gene(s) of interest, often tumor antigens, and are replication-defective (RD), which is a characteristic that aids in the safety of this treatment modality [23]. This is commonly implemented in vaccine development and has proven to provide effective protection with no serious adverse events such as clinical infection or shedding of the virus into the surrounding environment. This has been demonstrated in RD-recombinant chimpanzee adenovirus type 3-vectored ebolavirus vaccine (cAd3-EBO) and many other studies using adenoviral vectors as a vaccine platform [24].

From a biological perspective, DNA viruses demonstrate higher genome stability due to their high-fidelity DNA polymerases. Their larger genomes often allow for greater ability to incorporate larger transgene insertions without jeopardizing the capacity for viral infection and replication. Replication takes place in the nucleus. However, the large genome size impedes replication kinetics [25–27]. While DNA viruses can encode proteins that protect viral nucleic acid detection [27], these viruses are also able to a elicit strong antiviral responses which can aid in anti-tumor immunity. The caveat remains that high neutralizing antibodies (nAbs) may limit viral replication, thus hindering viral spread [28]; however there have been reports tjat oncolytic viruses are able to replicate efficiently even in the presense of nAbs that target the backbone virus [29].

On the other hand, RNA viruses such as Newcastle disease virus (NDV), poliovirus, and reovirus have limited genomic packaging capacity but can be more immunogenic. Some viruses may encode proteases that cleave RNA virus sensors, which inhibits the antiviral response [27]. Moreover, replication of RNA viruses takes place in the cytoplasm and demonstrate rapid proliferation. Their high mutation rates introduces new genentic variation due to the low-fidelity RNA polymerase [25,27]. This allows for rapid evolution toward a beneficital oncolytic phenotype but also may cause divergence from this desired characteristic. The issue of genetic stability has also been proposed as a possible advantage for “personalized” targeted therapy, where multiple optimized virus variants can promote tumor clearance even in the presence of antiviral immunity [30]. Thus, the use of RNA viruses can be a double-edged sword, thus calling for a judicious design in the construction of OVs and study designs.

Common examples of oncolytic DNA viruse include vaccinia virus (VV), adenoviruses, and herpes simplex virus (HSV). VV is a double-stranded DNA (dsDNA) virus that infects and replicates within the cytoplasm of mammalian cells [31]. There have been various vacccina virus agents being studied. Pexa-Vec is an oncolytic VV with inactivated thymidine kinase (TK) gene that is replaced with a transgene that expresses human GM-CSF and β-galactosidase [32]. Pexa-Vec has been evaluated in the treatment of hepatocellular carcinoma (HCC) and colorectal cancer [31,33,34]. Moreover, GL-ONC1 (VV with Ruc-GFP, β-glucuronidase, and β-galactosidase transgene insertions), vvDD (VV with deletion of the vaccinia growth factor and TK genes), and TBio-6517 (VV that expresses Flt3 ligand, the cytokine IL-12, and an antibody targeting CTLA4) are under clinical investigation [35–37].

Adenovirus is a dsDNA virus. Onyx-015 (lontucirev) was the first recombinant adenovirus to be tested in humans and features viral attenuation and conditional replication due to deletion of the E1B locus, which encodes for 55 kD E1B protein [38]. Onyx-015 has been discontinued midway through phase III trials looking at head and neck cancer in China despite the possible success in meeting the primary endpoint based on the interm report of efficacy and safety [39]. Second-generation adenoviruses such as DNX-2401 (tasadenoturev) have demonstrated success in treating glioblastomas [40]. The 24 base pair deletion of the E1A gene prevents DNX-2401 from replicating in cells that maintain normal retinoblastoma (Rb) pathways and selectively targets cancer cells with Rb pathway abnormalities [41]. DNX-2401 has shown success in treating malignant gliomas and has been granted fast track orphan drug designation by the US Food and Drug Administration (FDA) in malignant glioma in 2014 [40]. Currently, combination with immune checkpoint inhibitors are being pursued. Additional examples of oncolytic adenoviruses under clinical investication include enadenotucirev (chinerix Ad11p/Ad3 oncolytic adenovirus with a 25 bp deletion of E4 and 2444 bp deletion in E3ORF), LOAd703 (a serotype 5 adenovirus with serotype 35 fiber and knob and encodes trimerized membrane-bound CD40L and 4–1BBL), and ONCOS-102 (a modified sertotype 5 adenovirus with a serotype 3 knob, insertion of the GM-CSF transgene, and a 24 bp deletion of the Rb binding site of the E1A gene) [42–45].

Herpes simplex virus (HSV), specifically HSV-1 and HSV-2, is a dsDNA virus that naturally infects humans [46,47]. HSV oncolytic therapy has been applied to the treatment of melanomas, gliomas, and colorectal cancer [48,49]. HSV1716, a mutant that lacks the ICP34.5 neurovirulence gene, selectively targets and replicates in human glioblastoma cells [50]. NV1020 is a mutant HSV with deletions of a 15-kb region at the UL/S junction including the U_L_56 gene and further attenuation by a 700-bp deltion encompassing the TK gene and the U_L_24 promotor [51, 52]. Reinsertion of viral HSV-1 TK gene enables control of NV1020 infection with TK-converted prodrugs like acyclovir. Weekly hepatic arterial infusion of NV1020 was noted to stabilize the liver metastasis in 50% of patients with heavily treated colorectal cancer at the optimal biological dose of 1×10^8^ plaque-forming unit (PFU) [49]. Other HSV-based OVs that are under clinical trials include G207 (an HSV-1 strain with deletion of the neurovirulent _γ1_34.5 gene and insertion of β-galactosidase to inactiave U_L_39 gene), ONCR-177 (an HSV-1 agent with a mutant UL37 gene, tissue-specific miRNA attenuation, and insertion of five transgenes for IL-12, FLT3LG, CCL2, and antagonists against PD-1 and CTLA-4), OH2 (genetically modified HSV-2 which expresses GM-CSF), and RP1 (an HSV-1 agent that expresses GM-CSF) [53–57].

NDV is a single-stranded RNA (ssRNA) virus that naturally infects avian hosts (poultry) [58,59]. One of the most studied strains of NDV is MTH-68/H, which has been applied to treatment of epithelial tumors as well as high-grade glioma [48, 60, 61]. Another NDV agent, LaSota, is a lentogenic strain of lower pathogenicity. LaSota has been studied in vitro using HPV E6/E7 expressing TC-1 cells that serves as a cervical cancer model and showed that the tumor cells had suppressed growth by OV induced apoptosis [61].

Poliovirus is a ssRNA virus that naturally targets neurons, which makes this an effective vehicle for glioma-targeted oncolytic therapies. Poliovirus infection is limited to human and old-world primates due to viral binding with the poliovirus receptor Nectin-like molecule 5 (Necl-5) or CD155 in order to enter host cells [62,63]. Recombinant virus PVSRIPO is an attenuated chimera created from non-pathogenic strains of rhinovirus and type 1 poliovirus vaccine and has been studied in malignent glioma and melanoma [64,65]. The poliovirus internal ribosomal entry site (IRES) has been replace with that of rhinovirus [66]. Deletion of the poliovirus IRES attenuates neurovirulence and selects for conditional replication in tumor cells, specifically binding to CD155 which has been found to be highly upregulated in many cancer types [63,67].

Respiratory enteric orphan virus (reovirus) is a nonenveloped, double-stranded RNA (dsRNA) virus that is able to infect a wide range of mammalian hosts [68], including bats, humans, minks, and pigs [69]. Reovirus is mostly nonpathogenic in humans and has demonstrated preferential replication within cancer cells that express a constitutively activated Ras pathway. However, the virus does not affect nonmalignent cells without Ras activation [70]. Pelareorep is a shortened form of reovirus that was given an orphan drug status in 2015 by the FDA and the European Medicine Agency (EMA) for the treatment of malignant gliomas, ovarian cancer, and pancreatic cancer, which are considered as Ras-activated tumors [71]. Since then, reovirus has also been used to treat melanomas, breast cancer, and head and neck squamous cell carcinoma [72–74].

### Approved Oncolytic Viruses

The OV field is continuously gaining traction as a feasible option for immunotherapy, and intensive developmental piplines have led to the approval of four OVs throughout the world. The first registered OV was ECHO-7 (trade name Rigvir), which was approved in Latvia in 2004 [75]. ECHO-7 is a type 7, group IV, enteric cytopathogenic human orphan (ECHO) virus that has been repeatedly passaged in human tumor tissue cultures and selected for enhanced selective replication within tumor cells [75,76]. ECHO-7 was approved for local treatment of skin and subcutaneous melanoma metastases and delivered via intramuscular injections. However, it has been shown to be effective in a variety of cancer types other than melanoma, including colorectal, gastric, and small cell lung cancers [77,78]. Pumpure et al. (2020) documented the treatment of a female patient diagnosed with stage IVA primary malignant melanoma of the cervix. The patient reported no side effects or adverse reactions, and the patient had a survival of 67 months and progression-free survival (PFS) of 57 months at the time of publication [79]. However, the State Agency of Medicines of Latvia suspended marketing authorization of Rigvir in 2019 due to poor quality control [80].

In 2005, the Chinese State Food and Drug Administration approved H101 (trade name Oncorine) for treatment of head and neck cancer [81]. H101 is a type 5 recombinant human adenovirus with deletions of the gene that encodes the 55-kDa E1B protein and the E3 region gene segment. E1B works to bind and inactivate p53, thus deletion of this gene allows for proper p53 tetramer formation and cell cycle checkpoint regulation [82]. The E3 region contains seven expressed open reading frames that function to inhibit host immunity to enhance viral dissemination [83]. H101 has been tested on multiple types of solid tumors including gastric carcinoma, HCC, and lung cancer [84–86]. Zhang et al. (2021) evaluated H101 treatment with or without chemotherapy on 95 patients who were diagnosed with advanced gastric cancer. The study demonstrated that H101 combination therapy yielded a more effective response compared to single agent H101 or chemotherapy with a median overall survival (OS) of 29 months and a median PFS of 14.8 months [86].

In 2015, talimogene laherparepvex (tradename T-VEC) was approved as the first oncolytic virus by the FDA for local treatment of unresectable, cutaneous, subcutaneous, and nodal lesions of advanced melanoma or postoperative recurrent melanoma. T-VEC is a genetically modified herpes simplex 1 virus (HSV-1), where both copies of the gene that encodes infected cell protein 34.5 (ICP34.5), a peptide that enhances the virus’ neurovirulence [87], were deleted and replaced with a gene encoding GM-CSF. GM-CSF gene substitution induces secretion of the cytokine to recruit antigen presenting cells (APC) to the TME, and promote cytotoxic T lymphocytes (CD8+ T cells) responses to tumor-associated antigens (TAA). This modification is thought to improve viral replication in tumor cells that are defective in IFN pathways [88–91]. T-VEC has mainly been implemented in the treatment of melanomas. However, there has been some clinical trials focused on lymphomas as well [92,93]. Ramelyte et al. (2021) looked at intralesional T-VEC treatment of 13 patients with primary cutaneous B cell lymphomas (pCBCL). The patients reported mild side-effects such as flu-like symptoms, including chills, fever, and shivering, but no patients developed suspected HSV-associated systematic infection. T-VEC treatment demonstrated enhanced recruitment of an early innate immune response composed of activated natural killer (NK) cells and monocytes, followed by increased CD8+ T cell populations and reduced regulatory T cell (Treg) populations. Overall, T-VEC treatment was found to be effective in treating pCBCL (complete response (CR) = 46.2%, partial response (PR) = 38.4%, and progressive disease = 15.4%) [88]. A phase Ib trial investigated the T-VEC treatment in combination with Ipilimumab, a CTLA-4 inhibitor, in 19 patients with stage IIIB-IVM1c melalona that was not suitable for surgical resection. Puzanov et al. (2016) noted that the combination treatment was safe. A few patients developed grade 3/4 adverse events, but these findings did not lead to the discontinuation of T-VEC or ipilimumab. The treatment demonstrated promising results (CR = 22%, PR = 28%, stable disease (SD) = 22%). Probability of survival at 12 months and at 18 months was 72% and 67% respectfully [94]. Harrington et al. (2016) carried out a phase III OPTiM trial on the response rate of intratumoral injection of T-VEC compared to subcutaneous injection of GM-CSF in 249 patients with stage IIIB/C or IVM1a melanoma. OV treatment (Durable response rate (DRR) = 25.2%, overall response rate (ORR) = 40.5%) was determined to be more benefitial compared to GM-CSF treatment (DRR = 25.2%, ORR = 2.3%) Median OS of T-VEC versus GM-CSF treatment is 41.1 and 21.5 months respectively. Both therapeutic arms were well tolerated with patients reporting mild adverse events such as chills, fatigue, and influenza -like illness [95]. Thus, the data shows encouraging results suggesting that more in-depth research to confirm these results is warranted.

In 2021, teserpaturev (G47Δ; trade name DELTACT) was conditionally approved for malignant glioma in Japan. Teserpaturev is an HSV-1 with deletion of the both copies of the γ34.5 gene, and deletion of the α47 gene with the US11 promoter. The *lacZ* gene as inserted to inactivate the ICP6 gene [96]. The γ34.5 gene functions to impede host cell-induced shutdown of protein synthesis in response to viral infection. Thus, deletion of this gene allows for viral replication in cancer cells as malignant cells often lack the ability to inactivate protein synthesis [97]. Deletion of the α47 gene removes viral inhibition of host cell transporters associated with antigen presentation, leading to enhanced anti-tumor immune activation [98]. Lastly, inactivation of the ICP6 gene induces selective viral replication in actively dividing cells since ICP6 encodes the large subunit of ribonucleotide reductase that is needed for viral DNA replication [99]. Uchihashi et al. (2021) investigated teserpaturev treatment of oral squamous cell carcinoma in a murine model. Teserpaturev was found to inhibit growth of primary lesions and prolonged the survival of athymic nude and immunocompetent mice that injected with tongue cancer cells. Injected teserpaturev was found to immediately disseminate into cervical lymph nodes to effectively suppress lymph node metastases [100].

## Challenges of Oncolytic Virus Treatment

Implementation of OVs requires careful evaluation as there are multiple factors that must be taken into account. Different methods of inoculation have benefits and drawbacks with viral therapy. Moreover, the tumor extracellular matrix (ECM) must be accounted for as an important factor as well as the tumor stroma. Cell populations including cancer-associated fibroblasts (CAFs) and tumor-associated macrophages (TAMs) can dramatically hinder oncolytic virotherapy efficacy.

### Oncolytic Virus Administration

OVs can be administered either through direct inoculation into the tumor bulk or systemic injection, which includes intravenous (IV) or intraarterial (IA) injections [101]. There are benefits and challenges with both methods of administration. Direct intratumoral (IT) inoculation has been the most successful, as shown with FDA approved fast tracking of T-VEC. Direct IT inoculation maximizes the concentration of virus at the site of the lesion and thus induces a strong immunological response. However, this method is limited by tumor accessibility. Deep-seated tumors or those that are located in sensitive locations restrict the applicability and feasibility of IT inoculation as such invasive procedures carry a risk of injuries and complications. Moreover, other limitations include poor intratumoral retention due to viral dissemination into the bloodstream, limited viral dispersion in tumor tissues, and adverse inflammatory responses [102].

In contrast, systemic therapy utilizes the body’s vascular system to circulate OVs throughout the body similar to the delivery of chemotherapy or other anti-cancer agents. Likewise, there are a few hypothesized disadvantages associated with indirect inoculation. The first area of concern lies with systemic toxicity, whether the dosage of OVs may result in unanticipated off-tumor tissue or organ damage. Another major concern is immune clearance or the neutralization of OVs by the B cell generated antibodies, which interferes with internalization of the virus and dramatically abates the viral titer that ultimately reaches the tumor site [101,103].

This brings up the issue with seropositivity to the backbone virus, which is especially important viruses that are highly prevalent in the community. For example, there are multiple reports confirming high prevalence of seropositivity against human adenovirus (hAdV) infections throughout the world, including the United States, Australia, Japan, and the Philiphines. Ye et al. (2018) looked at the prevalence of nAbs to HAdV type 4 and type 7 in a group of volunteers from Hunan Province, China. The seropositivity rates for HAdV4 and HAdV7 nAbs were 58.4 and 63.8% respectively [104]. Thus, it can be predicted that a large portion of worldwide populations in areas with a history of HAdV infection contain high seropositivity for HAdV nAbs. The issue remains that seropositivity limits viral replication [105]. Neutralizing antibodies would bind to the OVs and inhibit cellular receptor binding [103,106]. Thus, decision about the choice of viral strain and the mode of administration should be made with careful consideration to preexisting immune responses.

Most of the literature agrees that suppression of humoral immunity is essential for systemic administrated oncolytic virotherapy [107]. The IFN pathway, specifically IFN-α, antagonizes OVs by reducing viral replication and stymying virus-mediated apoptosis [108]. Since cancers cells often lack a type 1 IFN response, these cell are more permissive to OV infection and replication [59].

Attempts have been made to protect OVs from the innate and adaptive immune system, specifically the humoral response with the use of IFN response inhibitors to enhance viral replication and efficacy of oncolysis. However, there have been safety concerns regarding the use of IFN antagonists. Saren et al. (2017) noted that treatment of glioblastoma bearing mice with Semliki Forest virus equipped with vaccinia virus-encoded type 1 IFN decoy receptor B18R controlled tumor growth but also induced severe neurotoxicity as the virus disseminated and replicated in healthy brain tissue [109].

Another method to protect OVs involves the use of genetically engineered protective coatings composed of chemical polymers, cell-derived nanovesicles, and liposomes that serve as a more direct method of overcoming the humoral immune response [110–112]. These protective coatings reduce immune recognition of the virus, thus limiting the production of nAbs against the OVs. The addition of tumor-targeting ligands can also help the OVs hone in on the tumor. The major concern with protective coatings is the practicality of the design. Protection of OVs increase the viral titer that reaches the tumor; however, the coatings may undermines the ligand-receptor interactions between OVs and tumor cell receptors resulting in reduced internalization of OVs. Moreover, additional drawbacks include issues with high production costs and limitations with large-scale transport of OVs [107].

Another feasible method is the use of carriers, either patient-derived cellular carriers (i.e., OV-infected cells that are injected back into the patient) or engineer carriers (i.e., nanoparticles). A wide range of cell types can be used as cellular carriers: endothelial cells, mesenchymal stromal cells, T-cells, and even tumor cells. However, there are safety concerns using certain cell types. Even though the patient’s own tumor cells are attractive from an immunologic standpoint, tumor cells or transformed cells should be studied with proper safety measurements. Furthermore, mesenchymal stem cells or neuronal stem cells demonstrate tumor tropism, allowing delivery of OVs throughout the body. However, such cell types are known to evade the immune system by allowing immune escape of tumor cells. The use of biodegradable nanoparticles is also gaining traction for compact delivery of viral antigens and the wide selection of nonmetal and metal-based compositions to maximize delivery of OVs [107]. Liposomal nanoparticles have demonstrated a high degree of biocompatibility with the host’s body and can be rapidly degraded by macrophages, making them a favorable candidate as a OV carrier [113].

### Challenges with Tumor Structure

Moreover, physical barriers such as the tumor stroma may prevent chemotherapy, tumor infiltrative effector cells, and OVs from effectively approaching tumor cells [114,115]. The tumor stroma is composed of non-tumor cells and structural components of the tumor tissue. Tumor cells are able to secrete cytokines to suppress certain anti-tumor functions of immune cells, while the stromal cells construct the desmoplastic stroma barrier, which physically impedes immune infiltration [116]. The stroma encapsulates the dense ECM, CAFs, TAMs, and tumor vasculature; all of which reinforce tumor resistance against the host’s immune system [117–119].

The ECM is generated by CAFs and poses the greatest barrier as it composes most of a tumor’s mass, creates an impenetrable barrier around the tumor, and undermines immune invasion and anti-tumor drug efficacy [119]. The denseness of the ECM also creates a paucity of oxygen and nutrients, which tumor cells exploit to induce activation of metabolic stress-related signaling pathways. Activation of these signaling pathways allows tumor cells to sculpt the TME to better suit their needs. For example, vascular endothelial cells (VECs) can dedifferentiate into tumor endothelial cells (TECs), which demonstrate enhanced proliferation, augmented migration capabilities, and facilitation of angiogenesis [120,121]. Another effect is the activation of drug efflux pumps and induction of senescence, both of which enhance tumor resistance against anti-cancer agents such as chemotherapy [119]. CAFs recruit myeloid-derived suppressor cells (MDSCs) and Tregs to create an immunosuppressive environment [122]. M2 TAMs have been shown to secrete TGF-β, which stimulates secretion and cross-linking of collagen, bolstering and fortifying the ECM [114,123].

Some studies have investigated methods to target the tumor stoma. For example, OVs expressing proteases such as matrix metallopeptidases (MMP)-9 can degrade ECM components. Sette et al. (2019) demonstrated that treatment of glioblastoma multiforme (GBM) with OV-derived HSV armed with MMP-9 increased viral invasion of GBM stem-like spheroids and improved survival of tumor-bearing nude mice [124]. In addition, OVs can be equipped with tissue inhibitor metalloproteinases 1–4 (TIMPs 1–4), which regulate proteolytic activity of MMPs and prevent rearrangement of the ECM [125]. Another method is to repolarize anti-inflammatory M2 TAMs into the pro-inflammatory M1 phenotype. M2 TAMs promote tumor proliferation through immune modulation and tolerance in addition to the recruitment of Tregs [126,127]. On the other hand, M1 TAMs secrete proinflammatory cytokines (e.g., IL-6, IL-12, and TNF-α) and reactive oxygen species (ROS) in order to enhance immune recruitment and function against malignant cells [128]. Rao et al. (2020) demonstrated the use of genetically engineered cell membrane-coated magnetic nanoparticles that triggers M2-M1 TAM repolarization demonstrated inhibition of tumor proliferation, reduced metastasis, and improved survival of mice with triple-negative breast cancer [129].

**Figure 2:**
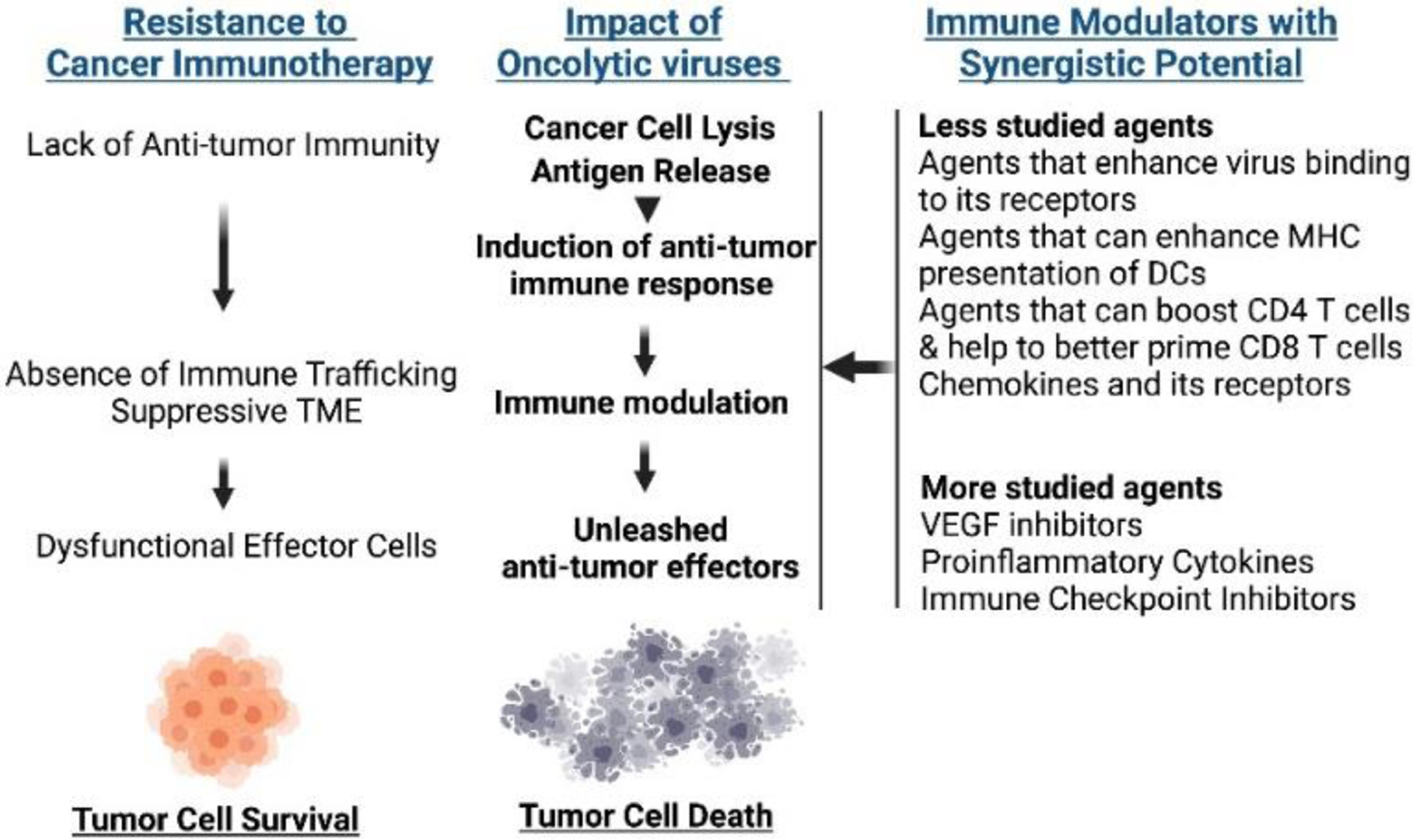
Strategies to Overcome Cancer Resistance to Immunotherapy using Oncolytic Viruses. Mechanisms of resistance to currently available immune checkpoint inhibitor therapies and other immunotherapy remain multifactorial. Oncolytic viruses have potential to help overcome primary or secondary resistance to immunotherapy independently or in combination with other immune modulatory agents.

Overall, the complexity of the tumor stroma and the various components work in tandem to create an immunosuppressive environment and a physical barrier against not only tumor infiltrating cells but also anti-cancer agents. OVs can influence the TME to convert the pro-tumor TME into an anti-tumor environment, but there still is room for improvement for the strategies described above. Figure 2 shows an overview of This highlights the need for novel approaches in OV development and more research in targeting both the tumor and the surrounding stroma.

## Current Oncolytic Virus-focused Clinical Trials

Given the compiled efforts in the field of oncolytic virotherapy, there are multiple ongoing clicical trials that investigate OVs in a variety of cancers, including breast, gastrointestinal, skin, and pancreatic cancers. The most common OV candidates include vaccinia virus, HSV, and adenovirus. A subset of ongoing clinical trials solely focus on determining patient response to OV single agent therapy, while the vast majority of trials use a combination approach (Figure 2), often pairing OV treatment with chemotherapy, monoclonal antibodies, or radiotherapy (Table 2, data was collected from clinicaltrials.gov in May 2022). However, there is a critical need for studies on accurate biomarkers to tailor and optimize therapeutic options that combine various treatments for specific patients as disease characteristic may differ across different patients.

Table 2 summarized the growing body of research that focuses on immune checkpoint inhibition (ICI) as a means to eradicate tumor cells as a combination partner with OVs [11,48]. Several monoclonal antibodies are developed to target immune checkpoints such as cytotoxic T-lymphocyte antigen 4 (CTLA-4), programmed death protein 1 (PD-1), and programmed death protein ligand 1 (PD-L1). PD-1 is essential in maintaining exhausted T cells and blocking PD-1 after the development of exhausted T cells can boost the T cell immune effector functions, which can disrupt tumor cell immune evasion [130]. ICI has changed the landscape of cancer care since the first FDA approval for anti-CTLA antibody ipilimumab in 2011. However, the majority of the patients do not benefit from ICIs as the overall response rate remains around 20–40% in most of studied regimens so far. Thus, overcoming primary resistance to ICI by offering an opportunity to induce both novel tumor antigen specific immune responses and innate immune responses while shifting the TME toward a pro-inflammatory state is appealing.

Ribas et al. (2017) studied the effect of oncolytic virotherapy, T-VEC, and pembrolizumab, an anti-PD-1 antibody, in patients with advanced melanoma. Previous studies have demonstrated that certain patients are resistant to PD-1 blockade due to the paucity of CD8+ T cells within the tumor lesion [131,132]. The use of T-VEC and anti-PD1 blockade combination elicited a strong immune response, increasing systemic circulation of CD4+ and CD8+ T cells, upregulated levels of T cell tumor infiltration, and reduced T cell exhaustion. Common T cell inhibitory markers include increased expression of CTLA4, PD-1, TIGIT, TIM3, and LAG3 [133]. The combination treatment demonstrated a reduction in tumor size with an overall response rate of 62%, and a CR of 33% in a phase 1b study (n=21) with low toxicity [22]. The phase 2 study (n=692) which was carried out in the same setting showed an acceptable safety profile but did not meet the PFS primary endpoint 14.3 (median; range = 10.3–22.1) months where the placebo and pembrolizumab arm showed PFS of 8.5 (median; range = 5.7–13.5, hazard ratio = 0.86; CI = 0.71–1.04, p = 0.13). The OS as a dual primary endpoint strategy is to be reported [134]. Overall, this strategy showed feasibility but requires further investigation into the most efficacious and synergistic combination regimen along with predictive biomarkers to better select the patients who will most benefit from the treatment with enhanced anti-tumor activity while minimizing unnecessary adverse events.

## Conclusions and Future Directions

OVs have come to the forefront of immunotherapy, offering a wide range of viruses as a backbone which can be genetically engineered to selectively target and replicate within tumor cells, while leaving normal cells unscathed. Ultimately, cell lysis releases various factors that attract immune cells toward the tumor while viral progeny infects neighboring tumor cells to continue the oncolytic cycle. The conditional replication of OVs make them an appealing therapeutic option. However, the administration of OVs must overcome various barriers such as viral neutralization by the humoral immune response and the hostile TME, which requires further investigation. Some studies have looked into using protective coatings or cellular carriers to overcome viral neutralization and enhance delivery of OVs to the tumor site. Lastly, a review of clinical trial registries for ongoing clinical trials in oncolytic virotherapy reflects a profound interest in the involved biomedical community especially in combination approaches with conventional cancer treatments such as surgery, chemotherapy, radiation, as well as novel immune modulators. Future studies need to verify the long-term safety and efficacy of incorporating OV therapy. Additionally, further research is needed to develop a strategy that can target cancer heterogeneity while ensuring proper receptor binding for viral entry in the setting of rapidly evolving cancer cells which may need to involve precision medicine to offer a more personalized approach for patients.

In summary, oncolytic virotherapy has secured its role to support cancer immunotherapy as the fourth pillar of cancer treatment, and research will continue to expand on the utilities of OV as an important element in multimodality approaches.

## Figures and Tables

**Table 1:** Comparison between DNA and RNA virus characteristics.

	DNA Viruses	RNA Viruses
Characteristics	Greater genomic stability	Genomic instability
	High fidelity DNA polymerases	Low-fidelity RNA polymerase
	Larger genomes	Smaller genomes
	Greater genomic packaging capacity	Limited genomic packaging capacity
	May or may not replicate in presence of neutralizing antibodies	
	Longer replication duration	
	Nuclear Replication	Shorter replication duration
		Cytoplasmic replication
	Mechanisms to block DNA virus sensing adaptors	Rapid evolution
		Mechanisms to block RNA virus sensing adaptors
Examples	Adenovirus	Echovirus
	Herpes Simplex virus	Measles virus
	Parvovirus	Newcastle disease virus
	Vaccinia virus	Poliovirus
		Reovirus
		Seneca Valley Virus
		Vesicular Stomatitis Virus

**Table 2:** Select ongoing clinical trials involving oncolytic virus and other anti-cancer therapies.

Identifier	Cancer Type	Phase	Oncolytic Virus	Injection	Cotreatment
NCT02705196	Pancreatic cancer	I/II	ADV	Intratumoral	Nucleoside, anti-PD1 Ab, &
					antimicrotubule agent
NCT03004183	NSCLC	II	ADV/HSV	Intratumoral	nucleoside, radiation,
	& breast cancer				& anti-PD1 Ab
NCT03916510	Rectal cancer	I	ADV	Intravenous	Radiotherapy
					& antimetabolite
NCT05051696	FG neoplasms	NA	ADV	Intratumoral	Radiotherapy
NCT05234905	FG neoplasms	II	ADV	Intratumoral	anti-PD1 Ab
NCT03252808	Pancreatic cancer	I	HSV	Intratumoral	Nucleoside &
					antimicrotubule agent
NCT03663712	Ovarian cancer	I	HSV	Intraperitoneal	NA
NCT03866525	GIC	I/II	HSV	Intratumoral	TOP1 inhibitor & anti-PD1 Ab
NCT04050436	SCSC	II	HSV	Intratumoral	anti-PD1 Ab
NCT04185311	Breast cancer	I	HSV	Intratumoral	anti-PD1 Ab, anti-CTLA4 Ab
NCT04349436	Carcinoma	I/II	HSV	Intratumoral	NA
NCT04755543	GIC	I	HSV	Intravenous	anti-PD1 Ab, alkylating agent, antimetabolites
NCT05232136	Bladder cancer	I/II	HSV	Intravesical	NA
NCT05235074	CNS tumors	I/II	HSV	Intratumoral	NA
NCT03043391	Glioma	I	Poliovirus	Intratumoral	NA
NCT03564782	Breast cancer	I	Poliovirus	Intratumoral	NA
NCT04445844	Breast cancer	II	Reovirus	Intravenous	anti-PD-L1 Ab
NCT02977156	Advanced cancer	I	VV	Intratumoral	anti-CTLA4 Ab
NCT03206073	CRC	I/II	VV	Intravenous	anti-CTLA4 Ab, anti-PD-L1 Ab
NCT03954067	Advanced cancer	I/II	VV	Intratumoral	w/wo anti-PD1 Ab
NCT04787003	Advanced cancer	I	VV	Intratumoral	w/wo anti-PD1 Ab, anti-PD-L1 Ab

1 Abbreviations: adenovirus (ADV), non-small cell lung cancer (NSCLC), herpes simplex virus (HSV), female genital (FG), gastrointestinal cancer (GIC), Topoisomerase 1 (TOP1), squamous cell skin cancer (SCSC), central nervous system (CNS), colorectal cancer (CRC), and Vaccinia virus (VV)
